# Examining Spillovers between Long and Short Repeated Prisoner’s Dilemma Games Played in the Laboratory

**DOI:** 10.3390/g9010005

**Published:** 2018-01-31

**Authors:** Antonio A. Arechar, Maryam Kouchaki, David G. Rand

**Affiliations:** 1 Department of Psychology, Yale University, Sheffield-Sterling-Strathcona Hall, 1 Prospect Street, New Haven, CT 06511, United States; 2 Programa de Estudios Longitudinales, Experimentos y Encuestas, Centro de Investigación y Docencia Económicas, Aguascalientes, AGS 20313, México; 3 Kellogg School of Management, Northwestern University, Evanston, IL 60208, United States

**Keywords:** cooperation, Prisoner’s Dilemma, repeated games, spillovers, dictator game, learning

## Abstract

We had participants play two sets of repeated Prisoner’s Dilemma (RPD) games, one with a large continuation probability and the other with a small continuation probability, as well as Dictator Games (DGs) before and after the RPDs. We find that, regardless of which is RPD set is played first, participants typically cooperate when the continuation probability is large and defect when the continuation probability is small. However, there is an asymmetry in behavior when transitioning from one continuation probability to the other. When switching from large to small, transient higher levels of cooperation are observed in the early games of the small continuation set. Conversely, when switching from small to large, cooperation is immediately high in the first game of the large continuation set. We also observe that response times increase when transitioning between sets of RPDs, except for altruistic participants transitioning into the set of RPDs with long continuation probabilities. These asymmetries suggest a bias in favor of cooperation. Finally, we examine the link between altruism and RPD play. We find that small continuation probability RPD play is correlated with giving in DGs played before and after the RPDs, whereas high continuation probability RPD play is not.

## 1. Introduction

Cooperation is central to successful human interaction, and research demonstrates that a key mechanism for promoting cooperation is repetition: when people interact repeatedly, the “shadow of the future” can make cooperation pay off in the long run [1]. In recent years, there has been considerable interest in examining experimental play in indefinitely repeated Prisoner’s Dilemmas (RPDs) in the laboratory [2–7]. This experimental work has indicated that, holding the stage game payoffs constant, cooperation tends to increase with the continuation probability—and, more generally, that cooperation increases with the extent to which the cooperative strategy “Grim”, which starts out cooperating but defects forever once one defect is observed, risk-dominates the non-cooperative strategy “Always Defect” [3,8].

Beyond asking how the game parameters affect cooperation within a setting, research has begun to ask how cooperation in one setting affects play in subsequent strategically-distinct settings—that is, how play under one set of incentives “spills over” to influence play under other sets of incentives. For example, Peysakhovich and Rand [9] found that participants who played a series of RPDs where “Grim” strongly risk-dominated “Always Defect” gave more in a subsequent Dictator Game (DG) compared to participants who played a series of RPDs where “Grim” was not an equilibrium. They explain this observation using the “social heuristics hypothesis” [10,11], which argues that people internalize rules of thumb for social interactions that prescribe behaviors that are typically payoff-maximizing. By this logic, RPD environments that incentivize cooperation lead participants to develop “habits of prosociality”, which then spill over to promote giving in the subsequent DG (and the opposite for RPD environments which incentivize defection). Further evidence of such spillovers comes from Stagnaro et al. [12], who find greater DG giving following a repeated Public Goods Game with centralized punishment relative to a Public Goods Game with no punishment. In both of these examples, behavior developed in a more socially- and strategically-complex game spills over to a subsequent simple one-shot anonymous allocation decision.

There is less evidence, however, regarding how play spills over between two equivalently complex interaction settings that differ in their strategic nature—for example, between RPDs with high versus low continuation probabilities. In this article, we directly investigate this issue by comparing cooperation in RPDs that transition from a long continuation probability where the cooperative strategy “Grim” is risk-dominant over “Always Defect” (i.e., an environment where we expect participants to learn to cooperate) to a short one where “Grim” is not an equilibrium (i.e., an environment where we expect participants to learn to defect), and vice versa.

We also examine the correlation between RPD cooperation and giving in DGs played before and after the RPD, seeking to replicate and extend the findings of Dreber et al. [13], who argue that social preferences only help to explain RPD play in the absence of cooperative equilibria. Finally, we ask whether play in the subsequent DG varies based on RPD condition and whether play in a game is related to the time participants take to reach a decision.

We do find some evidence of spillovers when participants switch from the long RPD games to the short RPD games, with relatively high cooperation in the initial rounds of the short games. However, cooperation quickly declines as participants adjust to the short games (and lack of cooperative equilibria). Interestingly, we observe the same cooperative transient at the beginning of the condition in which the short games are played first, suggesting that the transition from daily life to short RPDs is similar to the transition from long RPDs to short RPDs. Conversely, when switching from short games to long games, participants immediately begin to cooperate at a high level. We therefore observe some evidence of a bias in favor of cooperation. We also note that cooperation in the short RPDs declines to a lower level in the condition where short RPDs are preceded by long RPDs, compared to the condition where short RPDs are played first—perhaps because having just played long RPDs makes it more explicit/salient to participants that cooperation cannot be supported in short RPDs.

With respect to DG giving, we replicate Dreber et al. [13]’s finding that DG giving correlates positively with RPD cooperation in short games (where “Grim” is not an equilibrium) but not in long games (where “Grim” risk-dominates “Always Defect”); and we find that this is true for both the DG played before the RPD and the DG played after the RPD. We also find that giving in the post-RPD DG is lower than in the pre-RPD DG, regardless of the order of the long versus short RPDs.

We also explore the relationship between response time and decisions made in RPDs and DGs. We find that, on average, deciding to cooperate takes less time than to defect, and that participants take more time on average to reach a decision in the short RPDs. We also find that participants typically slow down when switching between conditions, except for those who gave in the DG and shifted towards an environment with long RPDs games, suggesting a bias in favor of cooperation among those participants.

The remainder of the paper is structured as follows. In Section 2, we review the related experimental literature. In Section 3, we introduce the experimental methods. In Section 4, we analyze our experimental results and their relationship with previous findings in the literature. Finally, in Section 5, we make concluding remarks.

## 2. Related Experimental Literature

Several researchers have investigated the role of time horizon on RPD play since the influential work by Rapoport and Chammah [14]. Notably, Roth and Murninghan [15] brought the concept of a stochastic continuation rule for repeated games to the lab and found more cooperative responses as the probability increased. Dal Bó and Frechette [3] investigated the evolution of cooperation and found that experience leads to repeated defection in environments where cooperation is not an equilibrium, but that in environments where cooperation *is* an equilibrium experience does not always lead to cooperation.

Dal Bó [2] compared finite and indefinite games with similar expected length and found higher cooperation rates in the latter. However, Normann and Wallace [16], and Lugovskyy et al. [17] with a different game and setup also compared finite and indefinite continuation rules and found no difference in terms of cooperation; on the other hand, Bigoni et al. [18], using a collection of continuous-time RPDs, reported higher cooperation rates with deterministic horizons. Overall, however, meta-analyses of Rand and Nowak [8] and Dal Bó and Fréchette [5] show that cooperation in infinitely repeated games increases with the continuation probability.

However, evidence of spillovers between two RPDs that vary their strategic nature is scarce. Duffy and Ochs [19] investigate the effect of switching from partner matching to stranger matching or vice versa (which is essentially the same as repeated to one-shot or vice versa). They show that, in fixed pairings (partner matching), a cooperative norm emerges as players gain more experience, and that such norm quickly disappears (albeit with a brief transient of cooperation) when switching to one-shot games without cooperative equilibria. Moreover, not only does a cooperative norm not emerge in treatments where participants are first matched randomly (stranger matching), but players engage in cooperative norms immediately after they switch to a partner matching environment.

Fréchette and Yuksel [20] modify the stage game payoffs of their RPD experiment such that cooperation is risk-dominant in the first part but not in the second. They also find brief cooperative transients when switching from games with cooperative equilibria to games without, but they do not vary the order in which these games are played.

Finally, evidence on cooperative transients or spillovers is also suggestive in other games. In an experiment on collusion in Bertrand oligopolies where participants are exposed to communication and no-communication conditions (or vice versa), Fonseca and Normann [21] find a *hysteresis* effect in which participants are much better at colluding without communication if this condition is preceded by the communication phase. Hence, collusion (i.e., cooperation) spills over from environments where communication is conducive to cooperation. Bednar et al. [22] also investigate behavioral spillovers by having participants play two distinct games simultaneously with different opponents. They find that, when participants play Prisoner’s Dilemmas in conjunction with Hawk–Dove games, participants cooperate less than when they play the Prisoner’s Dilemma alone.

## 3. Methods

Our workhorse for this study is the indefinitely repeated Prisoner’s Dilemma with stage game payoffs determined as follows: one Monetary Unit (MU) for mutual defection, four MUs for mutual cooperation, five MUs for defecting while the other cooperates, and zero MUs for cooperating while the other defects. We used a neutral labeling for the strategies in the experiment (“A” for cooperation and “B” for defection”) and participants were provided with the payoff information in every round. As can be seen Table 1, this is a standard two-player Prisoner’s Dilemma game with a static Nash equilibrium in mutual defection {“B”, “B”}.

To incorporate an indefinitely repeated nature into the experiment, we let participants continue playing for another round with the same partner with either a continuation probability of δ = 7/8 (average game length of eight rounds; “Grim” strongly risk-dominates “Always Defect” such that we expect participants to learn to cooperate) or δ = 1/8 (average game length of 1.14 rounds; “Grim” is not an equilibrium, such that we expect participants to learn to defect) [9].

### 3.1. Treatments

Our experimental manipulation was the order in which the games were played: either a series of δ = 7/8 games followed by a series of δ = 1/8 games (δ = 7/8 → δ = 1/8), or vice versa (δ = 1/8 → δ = 7/8). To avoid cross-treatment noise introduced by stochastic variations in game lengths and time allotted per session, we followed the procedure of prior work [4,6,7] and predetermined the number of rounds (50) and interactions (6 for δ = 7/8 and 21 for δ = 7/8) in each treatment according to their geometric distribution^1^.

In every interaction, participants were randomly assigned to play with another partner in the room. To avoid cross-session noise caused by different probabilities of meeting with another person, we restricted the number of participants to 12 per session.

### 3.2. Experimental Procedures

All the experimental sessions took place at the Kellogg School of Management between February and April 2016. The games were programmed in z-Tree [23], and a total of 168 people participated in 14 sessions (of 12 participants each)^2^. The average age was 20.7 years (±0.12 standard error of the mean (SEM)) and 60 percent were female.

At the start of each session, participants were randomly allocated to a cubicle in the laboratory and asked to play a one-shot Dictator game (DG1) with another participant in their session. Roles in the DG were assigned randomly and neither participant was informed of which role they had. Both players were asked how many MUs, out of 100, they would give to the recipient if they were assigned to be the Dictator. This allowed us to maximize the number of observations collected for the role of Dictator (instructions were presented on screen and a copy can be found in the Appendix).

Upon completion of the first task, participants received a printed copy of the instructions for the RPD (also included in the Appendix). The instructions were read out by the same experimenter through all the sessions and participants’ understanding was tested by having them individually answer a series of comprehension questions. To allow participants to familiarize themselves further with the game, the experiment proceeded with a one-shot trial interaction of the RPD with no consequences in terms of payoffs. Once this practice round was completed, the experimental treatments actually started with a collection of either 21 *short* interactions or six *long* interactions, totaling 25 rounds^3^.

A new set of instructions was administered right after the first part of the treatment had ended containing the details of the new games with a different continuation probability (either δ = 7/8 or δ = 1/8). Participants were informed about the existence of a second part to the experiment, but did not learn any of its details until the first part was completed. Similar to the first part, instructions were read out and a series of comprehension questions were administered, but this time no practice round was offered. Participants then played for another set of interactions that lasted for 25 rounds of combined play.

The session ended with a final round of the Dictator game (DG2), which was similar to the one administered earlier but played with a different participant in their session. Participants then completed a survey on their basic demographics and received payment for their participation in cash (the MUs earned throughout the session were converted into dollars at a rate of 30 MUs per dollar). A graphic summary of the experimental design is depicted in Table 2.

Overall, sessions lasted approximately 60 min and participants were paid an average of $19.22 (±0.08 SEM). Finally, statistical tests and analyses of RPD cooperation use logistic regression because it is a binary outcome, whereas analyses of DG giving and (log-transformed) response time use linear regression because it is a continuous outcome, both with robust standard errors clustered by session.

## 4. Results

### 4.1. Play in the RPD

Consistent with prior literature on play in indefinitely repeated games (for reviews, see [5,8]), we find that overall and first-round cooperation is significantly higher in δ = 7/8 than in δ = 1/8, regardless of the order played (*ps* < 0.01). Support for this result comes from Figure 1, where we evaluate how much participants cooperate in δ = 7/8 and δ = 1/8. When comparing the two δ = 1/8 conditions, we find relatively lower levels of cooperation when δ = 1/8 is introduced after δ = 7/8 (*ps* < 0.01), and no apparent order effects when comparing the two instances of δ = 7/8 (*ps* > 0.26).

We find evidence suggesting that participants learned to defect in δ = 1/8, and that they did not necessarily learn to increase cooperation rates in δ = 7/8. Figure 2 presents finer-grained results that focus on the temporal path of each of the environments and the order in which they are introduced. It reveals that, for both orders, cooperation in δ = 7/8 is already higher than in δ = 1/8 at the outset of the very first interaction (*ps* < 0.02), and that cooperation in the first round of the first interaction does not differ across orders for either δ = 7/8 (*p* = 0.49) or δ = 1/8 (*p* = 0.84). We also see that cooperation in δ = 1/8 has a negative trend over time regardless of order (*ps* < 0.01). Conversely, for δ = 7/8, cooperation did not change over time when δ = 7/8 was played first (*p* = 0.66), and even marginally increased over time when δ = 7/8 was played after δ = 1/8 (*p* = 0.08).

Notably, we observe a sharp increase in cooperation when transitioning from δ = 1/8 to δ = 7/8 (round-25 cooperation: 0.18, round-26 cooperation: 0.55; *p* < 0.01), but only a small decrease in cooperation when transitioning from δ = 7/8 to δ = 1/8 (round-25 cooperation: 0.48, round-26 cooperation: 0.40; *p* < 0.04). Hence, the evidence suggests that cooperative spillovers emerge when switching to games with a different continuation probability, and is in line with Duffy and Ochs [19,20].

To further investigate the dynamics of cooperation/defection in our experiment, we examine the strategies employed by the participants. We apply the “structural frequency estimation method” (SFEM) introduced by Dal Bó and Fréchette [3] to our data, and use the six strategies from their paper (Table 3): defect all the time (“Always Defect”); cooperate all the time (“Always Cooperate”); start out cooperating but defect forever once one defect is observed (“Grim”); start out cooperating and respond with what the other person did in the previous round (“Tit-for-tat”); start out cooperating and cooperate if either both cooperated or neither cooperated in the previous round, otherwise defect (“win-stay, lose-shift”); and start out cooperating, and if the other defects trigger two rounds of defection, after which cooperation is restored (“T2”).

We see that the vast majority of participants in δ = 1/8 play “Always Defect”, regardless of order. In δ = 7/8, participants are much more cooperative, and the majority use conditionally cooperative strategies. Interestingly, these results are qualitatively similar to previous evidence comparing games with continuation probabilities of δ = 1/4 and δ = 3/4 [3].

Finally, we note an important order difference in δ = 7/8: participants are more forgiving (i.e., more likely to play “Tit-for-tat” rather than “Grim”) after having previously played δ = 1/8 (δ = 7/8 first vs. δ = 7/8 second: “Grim”, *p* < 0.001; “Tit-for-tat”, *p* < 0.001). This may be because they have seen the costs of failing to coordinate on cooperation or because they are less angered by their partner defecting given that they themselves defected consistently in the earlier games, where δ = 1/8.

### 4.2. Play in the DG and Its Relationship with RPD

**Figure 3:**
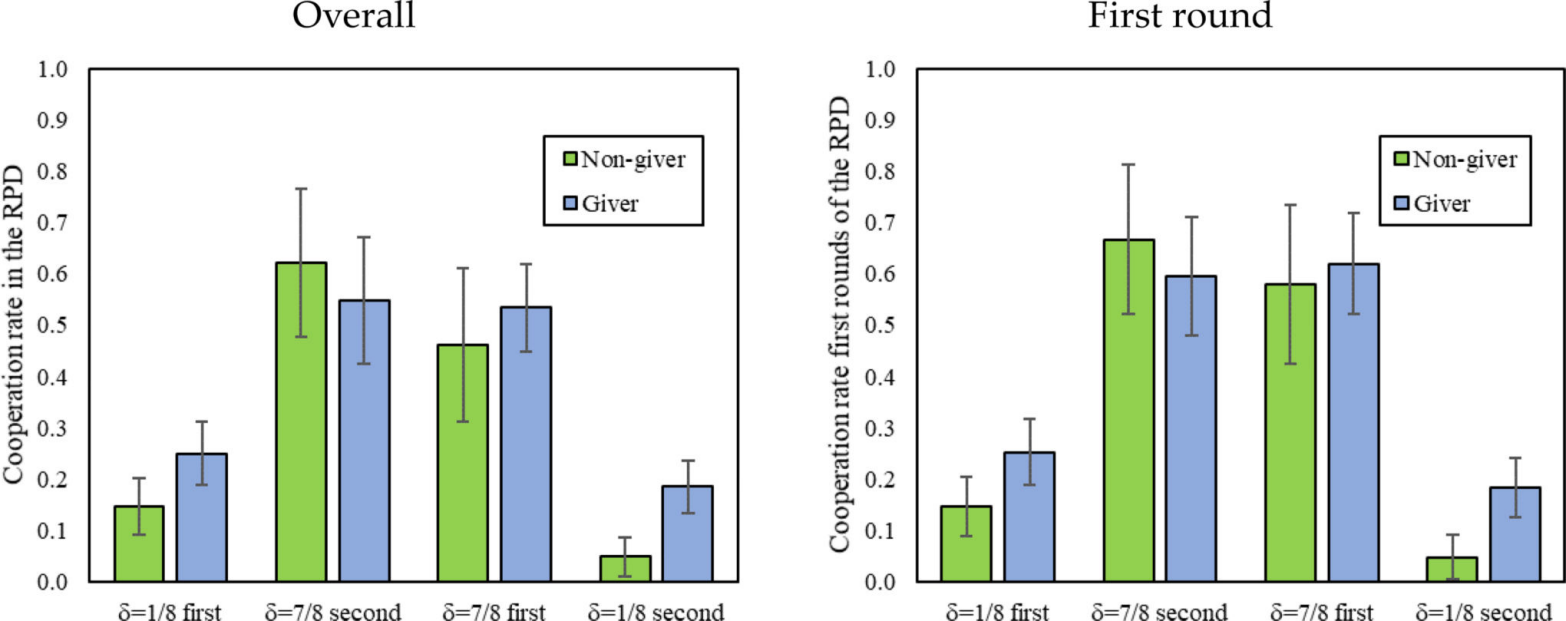
First and overall cooperation in the indefinitely repeated Prisoner’s Dilemma (RPD) of *givers* vs. *non-givers* in the Dictator game (DG1), by treatment. In addition, 95% confidence intervals clustered by session.

We now look at the level of cooperation in the RPD, conditional on the amount given in DG1 (the DG played prior to the RPD). To this end, we follow Dreber et al. [13] and divide participants into those who gave a positive amount as Dictators (*giver*, 70% of participants) and those who gave nothing (*non-givers*, 30% of participants). Figure 3 reveals that *givers* and *non-givers* cooperated equally in δ = 7/8, but that *givers* cooperated more than *non-givers* in δ = 1/8.

This is also confirmed statistically in Table 4 by a significant interaction between a δ = 1/8 dummy and a *Giver* dummy, and is consistent with previous findings by Dreber et al. [13] showing that only in environments without cooperative equilibria do *givers* tend to be more cooperative. Furthermore, we find no interactions with the order (whether δ = 1/8 or δ = 7/8 was played first). Thus, in the remainder of this analysis, we collapse decisions by order.

**Figure 4:**
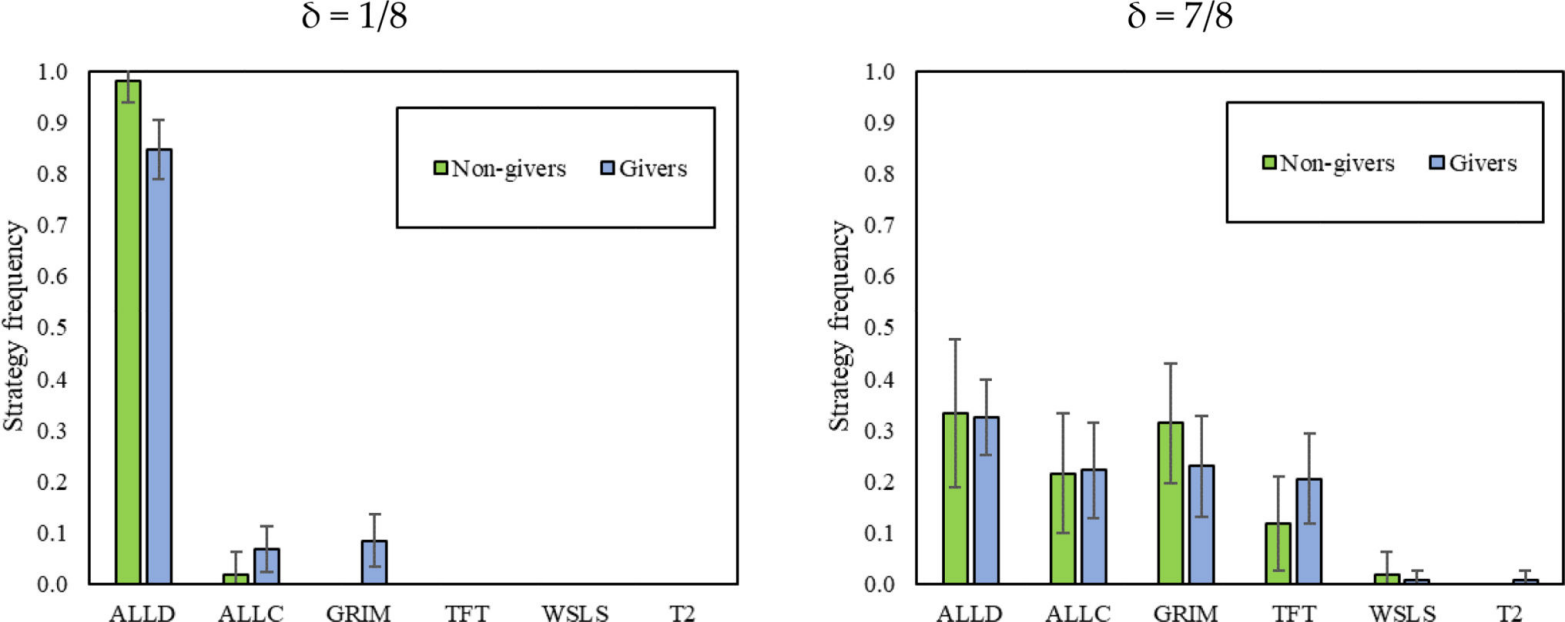
Strategy frequency of (*non-*)*givers* in the DG1. In addition, 95% intervals clustered by session.

Giving in the DG also predicts the strategies played in δ = 1/8 of the RPD. As Figure 4 shows, *givers* were less likely to choose “Always Defect” as their strategy in δ = 1/8, favoring “Grim” instead. Conversely, *givers* and *non-givers* do not seem to use different strategy sets when facing δ = 7/8.

Finally, we find no significant differences across RPD order in the amount sent in either the pre-RPD DG1 (δ = 7/8 first: 25.25 MUs; δ = 1/8 first: 24.62 MUs; *p* = 0.84) or in the post-RPD DG2 (δ = 7/8 first: 19.58 MUs; δ = 1/8 first: 18.95 MUs; *p* = 0.81). However, giving in DG2 was significantly *lower* than giving in DG1 for both treatments (*ps* < 0.01; −5.67 MUs decrease in both conditions).

### 4.3. Reaction Times and Play in the RPD and DG

We now explore the relationship between reaction times and play in the RPD. We find meaningful differences in terms of behavior, even though 92.50% of the decisions were made within three seconds and reaction times did not differ across treatments (1.43s in δ = 7/8 → δ = 1/8; 1.75s in δ = 1/8 → δ = 7/8; *p* = 0.59). In particular, participants took longer when choosing to defect than when choosing to cooperate (defect: 1.69s; cooperate: 1.40s; *p* < 0.01)^4^; they also took longer to decide in δ = 1/8 (1.36s in δ = 7/8; 1.81s in δ = 1/8; *p* < 0.01), but this difference was entirely driven by the δ = 1/8 → δ = 7/8 treatment (2.28s in δ = 1/8; 1.22s in δ = 7/8; *p* < 0.01).

**Figure 5:**
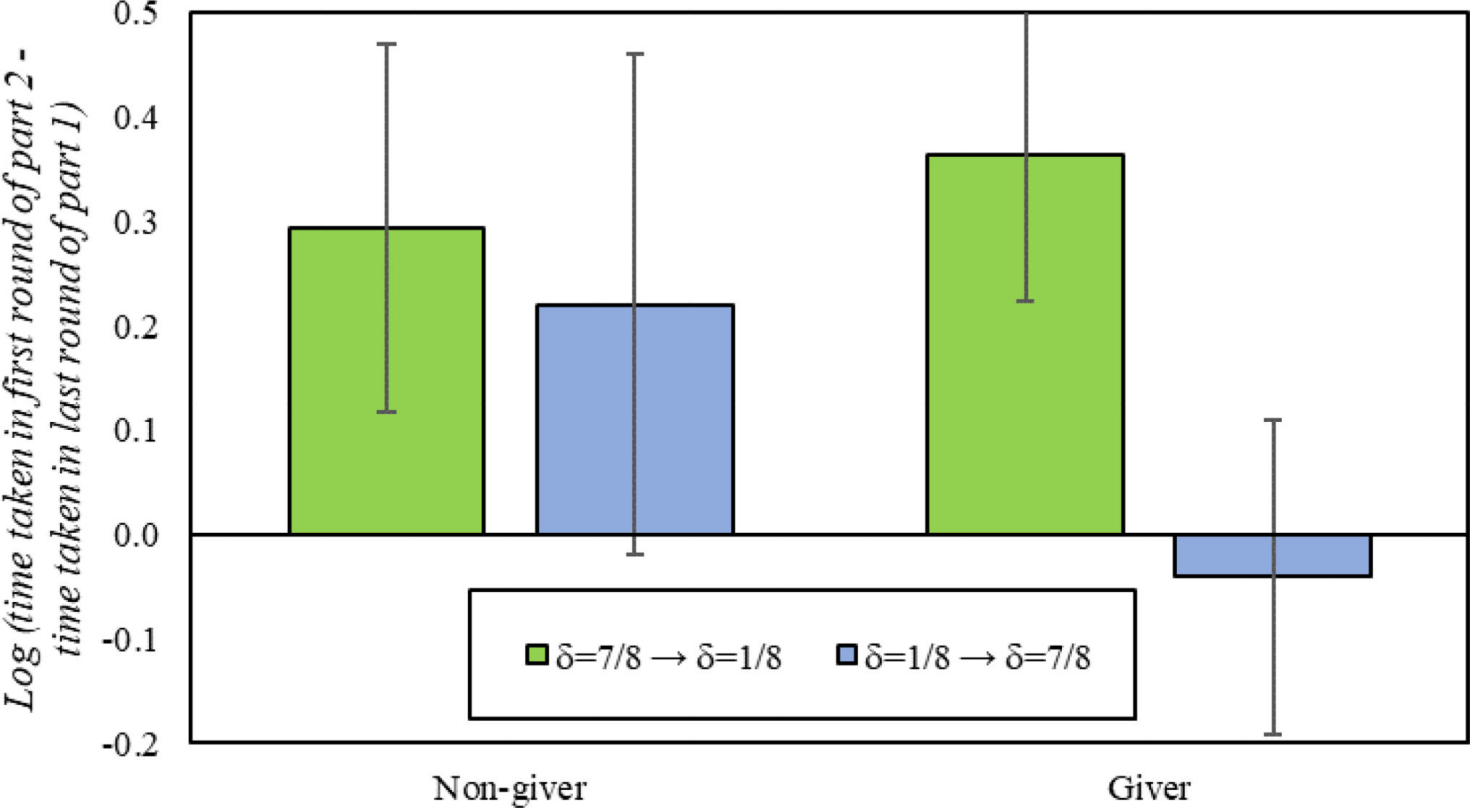
Difference in reaction times between the first round of the second part of the experiment and the last round of the first part of the experiment. In addition, 95% confidence intervals clustered by session.

With regards to play in the DG, givers and non-givers take statistically the same time to reach a decision (non-givers: 1.47 s; givers: 1.64s; *p* = 0.49). However, we observe some evidence in favor of cooperative spillovers when participants switch from one continuation probability to the other. Specifically, we find that participants typically slow down substantially when transitioning between conditions, except for givers switching into the δ = 7/8 condition, who do not slow down at all (*p* = 0.55; −0.32 s decrease; Figure 5). This suggests that givers in particular have a cognitive bias in favor of cooperation that causes them to respond more quickly than others when entering an environment that supports cooperation.

## 5. Discussion

This article investigated play in the indefinitely repeated Prisoner’s Dilemma—in particular the effects on cooperation of transitioning from a long continuation probability to a short one and vice versa, as well as the relationship between RPD play and Dictator Game giving.

Consistent with prior work on the RPD (e.g., [5,8]), we found substantially higher cooperation rates, and use of cooperative strategies, in the high continuation probability games where δ = 7/8 (such that “Grim” risk-dominates “Always Defect”) compared to the low continuation probability games, where δ = 1/8 (such that “Grim” is not an equilibrium). When transitioning from δ = 7/8 to δ = 1/8, we observe that cooperation remained somewhat high in the initial rounds of δ = 1/8, but then quickly declined with experience.

Interestingly, exactly the same pattern was observed when the δ = 1/8 games were played first—suggesting that coming into the lab and playing with δ = 1/8 involved making a transition from daily life (where δ is high) in the same way that playing δ = 1/8 after δ = 7/8 involves a transition. This pattern is indicative of a spillover effect: when people are used to cooperating (either outside the lab, or in the δ = 7/8 games), they continue to cooperate at a relatively high level when they first switch to δ = 1/8, and it takes several interactions for them to adjust. The fact that this happens to a similar extent in δ = 1/8 regardless of the order is at least consistent with the suggestion that δ = 7/8 is a reasonable model of the conditions participants experience outside the lab (and brought to bear at the outset of the δ = 1/8 to δ = 7/8 order). We do not have a clear explanation for why cooperation ultimately declines to a lower level in δ = 1/8 s compared to δ = 1/8 first, but it may be that making the transition from long to short games explicit leads participants to realize more fully that cooperation cannot be supported when δ = 1/8.

When considering δ = 7/8, conversely, we do not see much initial adjustment. Whether coming in from cooperating outside the lab or switching from a low level of cooperation in δ = 1/8, participants cooperate at a comparatively high level even in the very first round of δ = 7/8. Thus, there is an interesting asymmetry between cooperation and defection that is suggestive of a bias in favor of cooperation: when switching from δ = 7/8 to δ = 1/8, there is an adjustment lag in which participants initially try to cooperate before learning to defect, whereas no such lag occurs when switching from δ = 1/8 to δ = 7/8. This bias could be the result of an intuitive predisposition towards cooperation (e.g., [10,24]), or the result of explicit reasoning about, for example, efficiency.

Alternatively, our findings could also be interpreted there, being no spillovers whatsoever, and merely that participants play randomly at first, and then slowly transition towards their preferred choices as they gain more experience with the games—still, we observe some order differences that challenge this interpretation. For example, we identify a positive trend in cooperation when δ = 7/8 is played after δ = 1/8, but not when δ = 7/8 is played before δ = 1/8, and also note that cooperation in δ = 7/8 is consistently higher than in δ = 1/8 at the outset.

The strategy distributions found in this paper have some interesting parallels with the ones observed in Dal Bó and Fréchette [3], and provide additional insights. First, in both cases, defecting strategies describe the data better in the environments where cooperation is not an equilibrium. Second, in both cases, the cooperative strategy that is most often identified is “Tit-for-tat” (especially when δ = 7/8 is played after δ = 1/8). However, while “Grim” is not significantly present in [3], it is in our data (especially when δ = 7/8 is played first). In both cases, accounting only for “Always Defect”, “Grim” and “Tit-for-tat” can explain most of the data.

In line with Dreber et al. [13], we find that DG giving in the final stage of the experiment is correlated with cooperation in the δ = 1/8 environment, but not in δ = 7/8. Crucially, we also find the same relationship when considering giving in the DG played *before* the RPD. This shows that the correlation observed in [13] was not an artifact of the DG coming after the RPD (e.g., because of income effects). Instead, our results provide robust evidence that those whose social preferences drive them to give in the DG cooperate more in RPDs where cooperation is not an equilibrium. However, RPD cooperation in the presence of cooperative equilibria is not associated with social preference-based giving in the DG. That is, cooperation does not depend on social preferences in games where there is a self-interested reason to cooperate.

When comparing giving in the DGs played pre-RPD and post-RPD, we find that experiencing a combination of δ = 1/8 and δ = 7/8 reduces the degree of prosociality among participants, and that the order in which the environments are introduced does not affect such levels. This observation is potentially interesting in light of the evidence in Peysakhovich and Rand [9] suggesting that participants who experience an environment with δ = 7/8 are subsequently more prosocial than those who experience an environment with δ = 1/8. A possible explanation is that experiencing δ = 1/8 has a negative effect on prosociality beyond the positive one induced by δ = 7/8—that is, that “bad is stronger than good” [26]. Alternatively, the relative lack of positive spillovers in our study may have been due to the fact that the level of cooperation achieved in the δ = 7/8 condition of our experiment (roughly 55%) was much lower than in [9] (roughly 90%)—such that, even in δ = 7/8 in our experiment, a prosocial habit was not created.

Although the experiment was not designed to investigate reaction times, this work also identified some evidence suggesting a bias in favor of cooperation among altruistic players. Unlike participants who typically take long pauses to determine play in the first round of a new set of RPDs, altruistic participants (who gave in the DG) who transition to scenarios with long RPDs do not slow down at all. This may be the reflection of the time participants need to deliberate in (un)familiar scenarios: *non-givers* weigh up the potential benefits of strategic cooperation, regardless of the sequence of RPDs experienced; *givers*, on the one hand, hesitate to start cooperating in an environment where cooperation is not an equilibrium, but, on the other, they are ready to enlist in cooperation when the scenario is favorable.

Future work should clarify these observations through further exploration of spillovers between RPD play and subsequent DG giving, as well as spillovers from other types of institutions (such as centralized punishment, see [12]). In addition, new designs could also explore spillovers arising from environments where equilibrium selection is an issue. For instance, having participants play games with either low or high continuation probabilities in the first part of the experiment, and then play a constant stream of games that are conducive to stable cooperation in the second part. We would be surprised not to find spillovers in such scenarios.

## Figures and Tables

**Figure 1:**
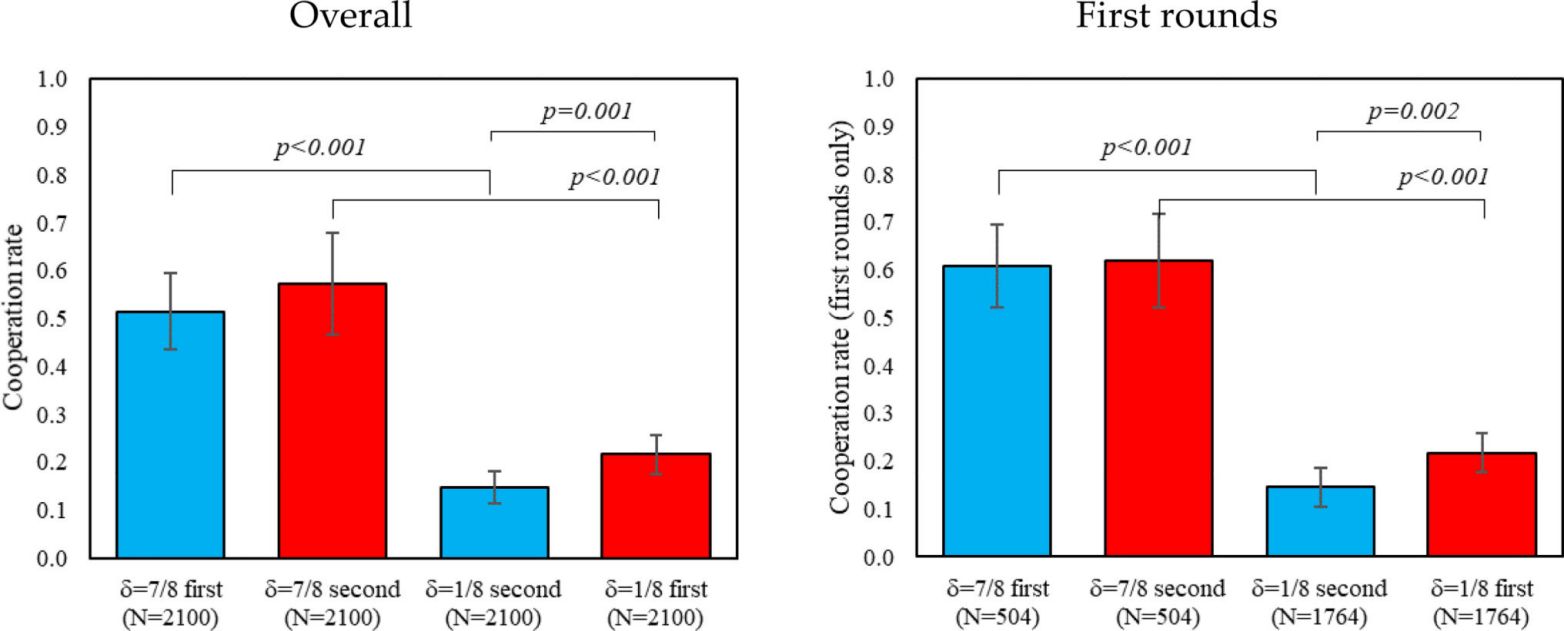
Difference in first rounds and overall cooperation by treatment. In addition, 95% confidence intervals clustered by session.

**Figure 2:**
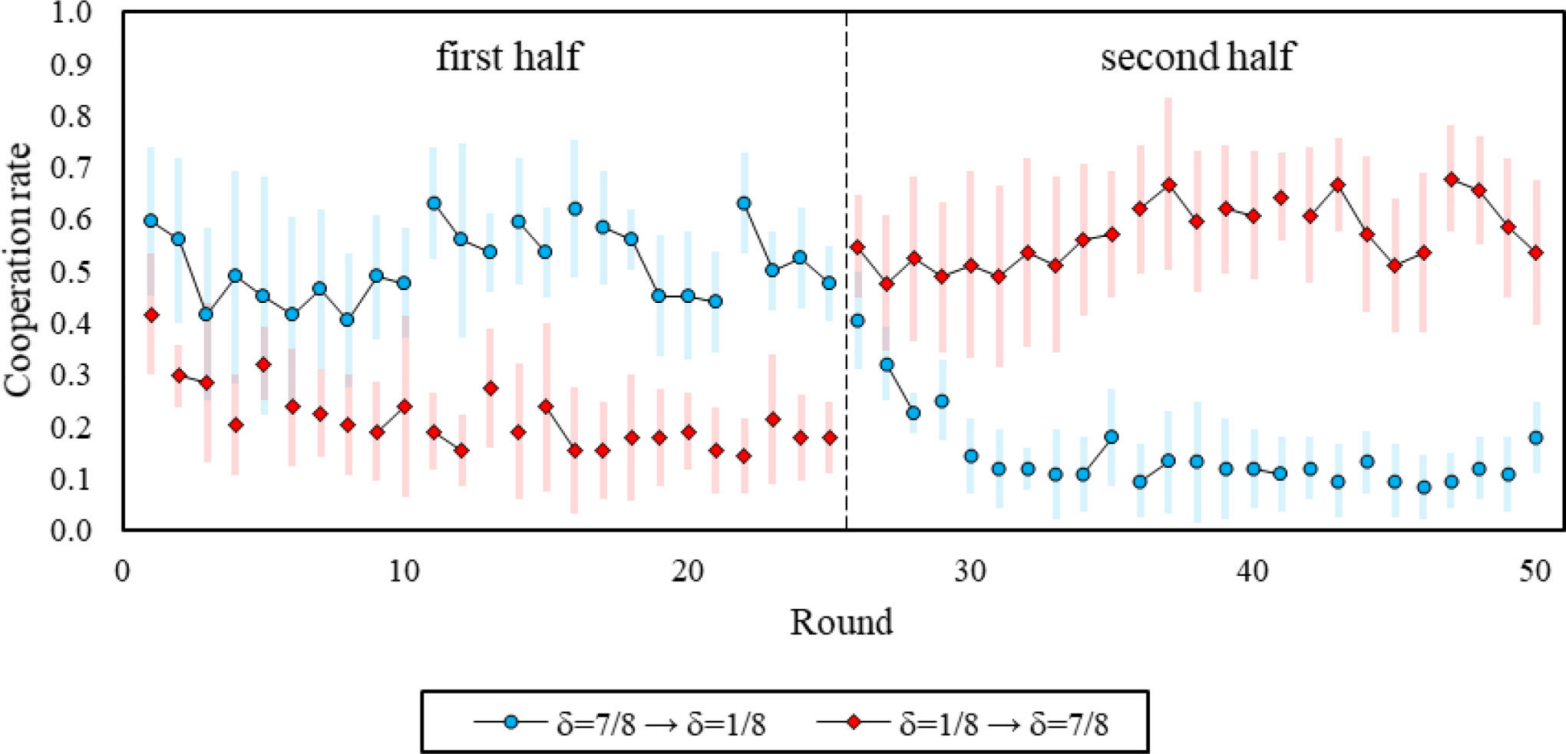
Cooperation over the course of the session, by treatment. Lines between dots represent rounds of a single interaction. In addition, 95% confidence intervals clustered by session.

**Table 1:** The stage game.

	Cooperate (A)	Defect (B)
*Cooperate (A)*	4, 4	0, 5
*Defect (B)*	5, 0	1, 1

**Table 2:** Timeline and experimental design.

Treatment			*Stage*			
			RPD			
δ=1/8 → δ=7/8; *n* = 84	DG1	Trial	21 *short* interactions; 25 rounds in total	6 *long* interactions; 25 rounds in total	DG2	Survey
δ=7/8 → δ=1/8; *n* = 84			6 *long* interactions; 25 rounds in total	21 *short* Interactions; 25 rounds in total		

Note: DG: Dictator game; RPD: Indefinitely repeated Prisoner’s Dilemma.

**Table 3:** SFEM results for each continuation probability, by order.

Strategy	δ = 1/8 first	δ = 1/8 second	δ = 7/8 first	δ = 7/8 second
Always Defect	0.89***	0.92***	0.35***	0.28***
	(0.04)	(0.03)	(0.06)	(0.05)
Always Cooperate	0.04	0.04	0.14***	0.13***
	(0.03)	(0.02)	(0.06)	(0.04)
Grim	0.07*	0.04*	0.26***	0.08*
	(0.04)	(0.02)	(0.08)	(0.04)
Tit-for-tat	0.00	0.00	0.23***	0.47***
	(0.01)	(0.00)	(0.07)	(0.07)
Win-stay, lose-shift	0.00	0.00	0.01	0.04
	(0.00)	(0.01)	(0.02)	(0.03)
T2	0.00	0.00	0.00	0.00
	(0.01)	(0.00)	(0.00)	(0.00)

Accuracy	0.83	0.88	0.85	0.88

Note: Accuracy is the fraction of moves correctly predicted by the strategy set. Bootstrapped standard errors in parenthesis; *p* < 0.10 *; *p* < 0.05 **; *p* < 0.01 ***.

SFEM: Structural frequency estimation method.

**Table 4:** Cooperation in the Prisoner’s Dilemma.

	Overall Cooperation			Round 1 Cooperation		

	*(i)*	*(ii)*	*(iii)*	*(i)*	*(ii)*	*(iii)*
Giver (in DG1)	−0.033	0.326	0.295	−0.081	0.264	0.169
	(0.198)	(0.228)	(0.236)	(0.206)	(0.241)	(0.276)

δ = 1/8	−2.364***	−2.679***	−2.815***	−2.699***	−3.148***	−3.317***
	(0.212)	(0.173)	(0.214)	(0.262)	(0.239)	(0.331)

δ = 1/8 first		0.694**	0.652*		0.503	0.372
		(0.321)	(0.337)		(0.317)	(0.359)

Giver × δ = 1/8	0.913***	1.040***	1.198***	0.971***	1.120***	1.328***
	(0.218)	(0.206)	(0.313)	(0.305)	(0.281)	(0.434)

Giver × δ = 1/8 first		−0.658**	−0.598*		−0.663**	−0.476
		(0.326)	(0.356)		(0.308)	(0.400)

δ = 1/8 × δ = 1/8 first		0.370*	0.563		0.596***	0.860*
		(0.214)	(0.369)		(0.226)	(0.464)

Giver × δ = 1/8 × δ = 1/8 first			−0.236			−0.342
			(0.406)			(0.557)

Constant	0.197	−0.179	−0.157	0.521***	0.253	0.322
	(0.188)	(0.235)	(0.236)	(0.180)	(0.239)	(0.250)

Pseudo R^2^	0.119	0.127	0.127	0.139	0.148	0.148

N	8400	8400	8400	4536	4536	4536

Notes: Logit regressions; standard errors clustered by session; *p* < 0.10 *; *p* < 0.05 **; *p* < 0.01 ***.

**FIGURE 1:**
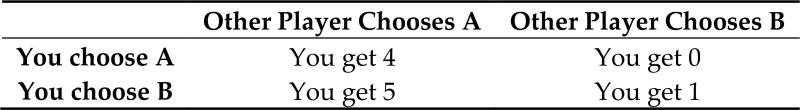
YOUR INCOMES

**FIGURE 2:**
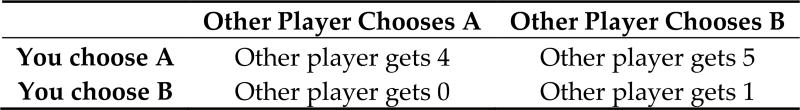
OTHER PLAYER’S INCOMES

## Footnotes

1 We recognize that a trade-off between equating the number of expected rounds in each set and the number of supergames emerges if the number of repetitions influences the participants’ experience. As there is no consensus in the current literature, we choose the former and set game lengths by interaction as follows: 10, 3, 2, 1, 5, 4 in δ = 7/8, and 1, 2, 1, 1, 1, 1, 1, 2, 2, 1, 1, 2, 1, 1, 1, 1, 1, 1, 1, 1, 1 in δ = 1/8. Yet, if the relevant unit of learning is the supergame, then participants in our experiment undergo more learning in the δ = 1/8 condition than the δ = 7/8 condition.

2 All subjects gave their informed consent for inclusion before they participated in the study. The study was conducted in accordance with the Declaration of Helsinki, and the protocol was approved by the Ethics Committee of ****** University (STU00202085).

3 We do not find evidence of spillovers from the practice round to the first round (i.e., participants’ choices in the practice round do not differ significantly from the first round of the first interaction of actual play, *ps* > 0.22), and thus the results in the next session do not include data from this non-incentivized trial.

4 This result has been widely documented in other games and scenarios (for reviews, see [24,25]).

## Appendix

### Experimental Materials

#### II. Instructions for the Indefinitely Repeated Prisoner’s Dilemma (δ = 7/8 → δ = 1/8 Order; first part)

Please read the following instructions carefully. If you have any questions, do not hesitate to ask us. Aside from this, no communication is allowed during the experiment.

##### Instructions

This is a computerized experiment on decision-making. You will be paid for participating and the amount you earn will depend on the decisions that you make.

The full experiment should about 60 minutes. At the end of the experiment, you will be paid privately and in cash for your participation.

All information collected in this experiment will be anonymous and neither the experimenter nor other participants will be able to link your identity to your decisions. In order to maintain this privacy, please do not reveal your decisions to any other participant.

We consider ourselves bound by the promises we are making to you in this protocol, we will do everything we say and there will be no surprises or tricks. We are interested in individual choices so please **remember that there are no right or wrong answers**.

##### Payment

In this experiment, you will earn Monetary Units (MUs) through the decisions that you make. At the end of the experiment, these MUs will be converted into dollars at a rate of 30 MU per dollar.

In addition to any money you earn from your decisions, you will also receive a $10 show-up fee.

##### The Experiment

The experiment will be split into two parts, Part A and Part B. *Your decisions in Part A will not at all affect what will happen in Part B*. You will be able to earn MU in both parts and your final income will be the sum of the MU you get in Part A and Part B. The instructions for Part B will be given on your computer screen after the completion of Part A.

##### Part A

Part A will consist of many individual sub-sections called *interactions*.

At the beginning of each *interaction* you will be matched with another individual in the room. You will then play a random number of *rounds* with this individual.

In each *round*, you will both be presented with two options: **A** and **B**. You will both receive an income in MU at the end of each round. This income will depend on both the choices that you and your opponent have made.

The figures below show how your incomes in a round will be determined:

For example:

If you choose A and the other person chooses A, you would both get 4 units.
If you choose A and the other person chooses B, you would get 0, and they would get 5 units.
If you choose B and the other person chooses A, you would get 5 units, and they would get 0 units.
If you choose B and the other person chooses B, you would both get 1 unit.

*You must make your choice within 30 seconds, if you do not, the computer will make a random choice for you for that round*.

Your income for each round will be calculated and presented to you on your computer screen. This income will be added to your current stock of MU.

Remember that the total number of MU you have at the end of the session will determine how much money you earn, at an exchange rate of 30 units = $1.

##### Rounds per Interaction

After each *round*, there is a 7/8 probability of another *round*, and 1/8 probability that the *interaction* will end. This probability does not depend on how many rounds you have already played.

Once the interaction ends, you will be randomly re-matched with a different person in the room for another interaction. Choices that you make will not influence either the number of interactions you have or the number of rounds in any interaction.

##### Summary of Part A

To summarize: every *interaction* you have with another person in the experiment includes a random number of rounds. In each *round*, both you and the person you are matched with will make one decision, this decision will determine your incomes for that round.

In every interaction, after every *round*, there is a 7/8 probability of another *round*. There will be a number of interactions, and your behavior has no effect on the number of rounds or the number of interactions.

At the beginning of the experiment, you will start with some MU in your account. You will earn MU in every round of every interaction. At the end of the experiment, you will receive $1 for every 30 MU in your account.

You will now take a very short quiz to make sure you understand the setup.

Part A will begin with one practice round. This round will not count towards your final income.

**Table:**

QUIZ	
If you choose **A** and the other person chooses **B** you will receive…	a. 0 units
	b. 4 units
	c. 1 unit

If you choose **B** and the other person choose **A**, you will receive…	a. 5 units
	b. 1 unit
	c. 0 units

The number of rounds in an interaction depends on your actions in that interaction or other interactions.	TRUE
	FALSE

If you have already played 2 rounds, the probability that there will be another round in your interaction is…	a. 4/8
	b. 7/8
	c. 0
	d. 1/8

If you have already played 5 rounds, the probability that there will be another round in your interaction is…	a. 4/8
	b. 7/8
	c. 0
	d. 1/8

*If you have any questions, please ask right now. Aside from this, no communication is allowed during the experiment*.

#### III. Instructions for the Indefinitely Repeated Prisoner’s Dilemma (δ = 7/8 → δ = 1/8 Order; second part, announced and presented right after the first part was completed)

In this part, after each *round*, there is a 1/8 probability of another *round (with the same player)*, and 7/8 probability that the *interaction* will end. This probability does not depend on how many rounds you have already played.

Once the interaction ends, you will be randomly re-matched with a different person in the room for another interaction. Choices that you make will not influence either the number of interactions you have or the number of rounds in any interaction.

You will earn MU in every round of every interaction. At the end of the session, you will receive $1 for every 30 MU in your account.

In this part, in every interaction, after every *round*, there is a 1/8 probability of another *round*. There will be a number of interactions, and your behavior has no effect on the number of rounds or the number of interactions.

You will now take a very short quiz to make sure you understand the setup for this part.

**Table:**

QUIZ	
The Number of Rounds in an Interaction Depends on Your Actions in That Interaction or Other Interactions	TRUE
	FALSE

If you have already played 2 rounds, the probability that there will be another round in your interaction is…	e. 4/8
	f. 7/8
	g. 0
	h. 1/8

If you have already played 5 rounds, the probability that there will be another round in your interaction is…	e. 4/8
	f. 7/8
	g. 0
	h. 1/8

If you have any questions, please ask right now. Aside from this, no communication is allowed during the experiment.
